# Titration of spoilt beer samples by flow-injection analysis

**DOI:** 10.1155/S1463924682000443

**Published:** 1982

**Authors:** J. G. Williams, M. Holmes, D. G. Porter

**Affiliations:** Laboratory of the Government Chemist Cornwall House Stamford Street London SE1 9NQ UK


Journal of Automatic Chemistry, Volume 4, Number 4 (October–December 1982), pages 176–182

### Titration of spoilt beer samples by flow-injection analysis

**J. G. Williams, M. Holmes and D. G. Porter**

*Laboratory of the Government Chemist, Cornwall House, Stamford Street, London SE1 9NQ*

### Introduction

In the UK, beers that are spoilt during manufacture, or in the course of distribution to the public, are the subject of repayment of excise duty. Samples of the spoilt beers returned from the distribution network to the brewery of origin are submitted to the Laboratory of the Government Chemist (LGC) for a determination of original gravity, in the course of this it is necessary to measure the acidity in order to apply a correction for any alcohol converted by microbiological oxidation to acetic acid. An inexpensive flow-injection system has been designed at LGC for automating the colorimetric titration of spoilt beer samples.

Flow-injection analysis (FIA) has been defined as the automated or semi-automated analytical process whereby a sample solution is inserted into a continuously flowing unsegmented stream (carrier stream) with subsequent detection of the analyte. The carrier stream serves as a reactor for the injected samples. By varying the dimensions of the tubing which accommodates the carrier stream, and by appropriate selection of pumping speeds and sample volumes, a wide range of concentration gradients of the sample within the stream is obtainable. Without air segmentation, the variation of concentrations with increasing distance downstream from the point of injection can be highly reproducible. Unlike air-segmented continuous-flow systems of the 'AutoAnalyzer' type, where the air bubbles limit the dispersion of the sample zone, the unsegmented FIA system is capable of greater flexibility.

The method described here is an application of high-dispersion flow-injection analysis [1]. FIA is now a well-established technique and it is not considered necessary to describe its development in detail. It is sufficient to refer to the excellent review of the early history of FIA by Stewart [2]. The principles and theoretical aspects of titrations by FIA have been described by Ramsing *et al.* [3]. Pardue and Fields [4] recorded a quantitative evaluation of flow-injection systems employing a gradient chamber with the aid of physical models. They used the models to compute concentration versus time profiles.

The technique of using FIA for the analysis of spoilt beers relies on the highly reproducible concentration gradients formed between an injected sample zone and a carrier stream. Other methods described in the literature rely on measuring the span between points of identical gradient dispersion [5]. The FIA method described in this paper relies on measuring the time span between points of inflection of the sigmoidal curves corresponding to the advanced and trailing gradients of the sample zone.

The method was designed to replace a manual acid/base titration which had been performed at LGC for the past 20 years. The volatile acidity of beer samples is found by the difference between total and 'residual' or non-volatile acidity, both are determined by titrating with 0·1 M sodium hydroxide using phenolphthalein as indicator [6].

Problems arise with the manual titration due to the difficulty of determining the end-point in samples varying widely in colour because of differences in caramel content. This has been overcome in the automatic flow-injection method.

The system uses software control of sampler and injection sequencing, as well as data handling by a PET microcomputer and simultaneous linear-regression evaluation. The transmittance/time analogue output from a colorimeter approaches in the ideal two sigmoidal curves back to back. Each sigmoid results from the interaction of an alkaline stream containing phenolphthalein with the interfacial regions of a slug of sample previously injected into a water carrier stream. One sigmoid curve therefore represents a titration at the advanced interface whilst the other sigmoid represents a titration at the trailing interface. The distance separating the points of inflection of each sigmoid is proportional to the logarithm of the hydrogen ion concentration of the sample. A circuit initiates and terminates monitoring by a PET of the real-time separation of the inflection points. The circuit employs both the first and second derivatives of the change of tramsmittance with time to control the output from an 'AND' gate in order to initiate and terminate timing.

### Experimental

### *Apparatus*

The components of the flow-injection system are bolted to a piece of Arborite (obtainable from Central Plastics, London), itself supported in a metal frame of Speedframe (made by Dexion Ltd, Wembley, Middlesex, UK) so as to produce a unit of laboratory equipment excluding the detector, sampler and reagent bottles.

The system consists essentially of two flowing streams. One is the sample stream which originates at a sampler probe and initially conveys either the sample or the water wash to waste. The second stream consists of a water carrier stream which flows to a colorimetric detector. The two streams interact via a four-way pneumatically activated Cheminert stream sampling valve. The sampler is a Technicon sampler (type II, obtained from Advanced Medical-Supplies, Aldershot, Hampshire, UK) which was modified by removing the cam timer mechanism. The sequencing of the sampler is controlled by the PET: this arrangement allows the independent control of sample and wash times. The switching relay contacts of the sampler are synchronized to the switching of two Martonair DM/1400/MY (Martonair Ltd, Twickenham, London) solenoid air valves. The air valves in turn control the two pneumatic actuators of the Cheminert valve (Laboratory Data Control, Stone, Staffordshire, UK) (see figure 1). When the sampler is in the sampling mode the sample stream follows a pathway around the largest of the two sampling loops of the stream sampling valve, whilst the water carrier stream flows independently around the smaller of the two sampling loops. When the sampler switches from sampling mode to wash mode, the effect is to interchange the contents of the two sampling loops so that a slug of sample is introduced into the carrier stream. Accurate sequencing of sampling and wash times is important to ensure that the slug of

 © Crown Copyright 1982

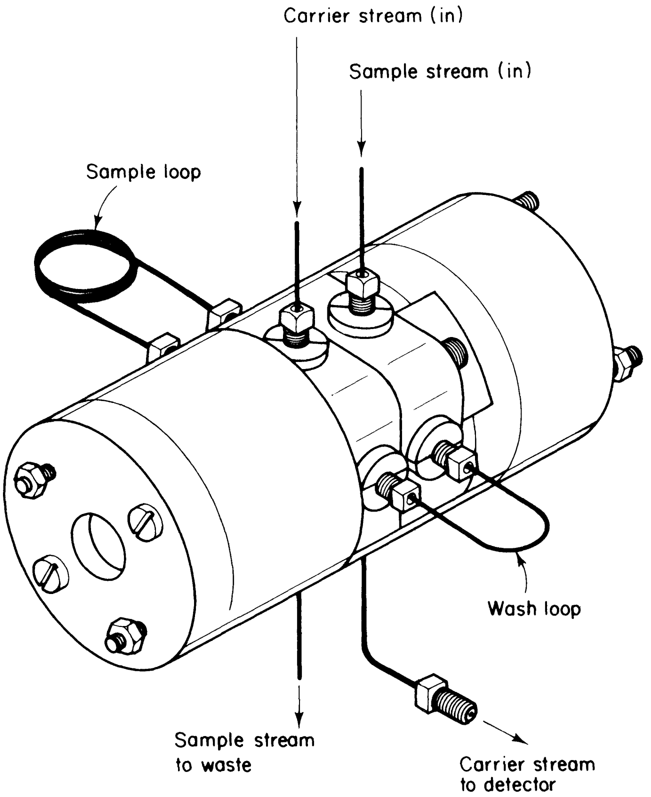

*Figure 1. Four-way pneumatically activated stream-sampling valve.*

air, introduced when the sampler probe is removed from the solution, has sufficient time to travel around the loop to waste before injection into the carrier stream.

After injecting the sample as a slug into the carrier stream, the sample passes to a Perspex dispersion chamber where a rotating magnetic follower aids the dispersion of the sample within the carrier stream. The concentration gradients at the interfaces of the sample slug with the carrier stream are continuously titrated with a stream of sodium hydroxide containing 0·02 g/l of phenolphthalein. The carrier stream them passes with minimum further dispersion to a Fison's Vitatron colorimeter where the transmittance at 550 nm is measured. Sampling rates of 65/h are typical. All pumping is carried out using a six-roller Ismatec mini-S-6 peristaltic pump (obtainable from Frost Instruments Ltd, Wokingham, Berkshire, UK) using standard AutoAnalyzer pump tube. The flow cell has a volume of 80 $\mu$l and an optical path length of 1 cm.

### Reagents

Analyses are normally performed by preparing 10 standards of hydrochloric acid with acidities ranging from 1 to 3·2 pH units. The standards, carrier stream and titrant are all prepared from freshly distilled water. The titrant is 0·0005 M sodium hydroxide containing 0·02 g/l of phenolphalein and stored in a polythene bottle fitted with a carbon dioxide trap containing 'Carbosorb'. A similar carbon dioxide trap is used on the reservoir of the carrier stream. Figure 2 shows the pumping arrangement of the system, and figure 3 the microcomputer control. All tubing, apart fron the pump tubes, is 1·02 mm internal diameter poly(tetrafluorethene). The sampling stream and carrier are both pumped at 5 ml/min whilst the sodium hydroxide stream is pumped at 3 ml/min. The 10 standards are prepared from a stock solution of standard 1 M hydrochloric acid by dilution with freshly distilled water according to the following routine:

(*a*) 20 ml of 1M HCl standard solution diluted to 200 ml (0·1M).

(*b*) 7·5 ml of 1M HCl standard solution diluted to 100 ml (0·075M).

(*c*) 5 ml of 1M HCl standard solution diluted to 100 ml (0·05M).

(*d*) 2·5 ml of 1M HCl standard solution diluted to 100 ml (0·025M).

(*e*) 20 ml of solution *a* diluted to 200 ml (0·01M).

(*f*) 7·5 ml of solution *a* diluted to 100 ml (0·0075M).

(*g*) 5 ml of solution *a* diluted to 100 ml (0·005M).

(*h*) 2·5 ml of solution *a* diluted to 100 ml (0·0025M).

(*i*) 10 ml of solution *e* diluted to 100 ml (0·001M).

(*j*) 7·5 ml of solution *e* diluted to 100 ml (0·00075M).

Other standards were prepared in a similar manner but using varying proportions of sucrose and caramel to study the effects of viscosity and colour density. Several beer samples were selected and the percentage transmittance measured at 550 nm. A typical value was used to prepare standards of constant acidity but of varying sucrose concentrations. Similarly, solutions with constant sucrose concentrations and acidities but of different caramel concentrations were prepared.

### Operation of point of inflection detector

At the point of inflection the first derivative of the signal is a maximum or a minimum and the second derivative is zero. Input B1 of the AND gate (figure 4) is the output of a comparator which compares the second derivative of the signal with zero. When the signal passes through a point of inflection, B1 will change from low to high or vice versa.

This alone is not sufficient to give an indication of a point of inflection, since when the gradient of the signal is zero the second derivative is also zero giving an undefined output from the comparator (see figure 5).

To avoid this, the comparator is logically 'ANDed' with the output from a 'zero voltage' detector which gives a high output only when the first derivative is greater than $\pm 1{\cdot}8$ V. Thus the output of the AND gate will only change when the second derivative passes through zero and the first derivative is above or below a certain level.

The output is connected to the user port of the PET where the width of the peaks are measured using software.

### PET software (*figure 6*)

The program has three functions: control of the sampler and valves; measurement of the width of the peaks; and a statistical analysis of these width measurements.

After detection of the rising edge of the peak the program runs in a loop between lines 40 and 53. An internal clock (ti$), which is reset at the beginning of each sample, is used for timing. When ti$ reaches set times d1$ and d2$, the sampler and valves are operated via an interface connected to the parallel user port. When ti$ > d3$ the program breaks out of the loop and sets the peak width *ti* (*N*) to the time between the first and the last point of inflection detected. The program then returns to line 15 to detect the start of the next peak. If the required number of peaks has already been detected then the program starts the linear regression analysis of the data. The first 10 values *ti* (*N*) are for the standards. A linear regression analysis is performed on these values together with the 10 concentrations in the DATA statement.

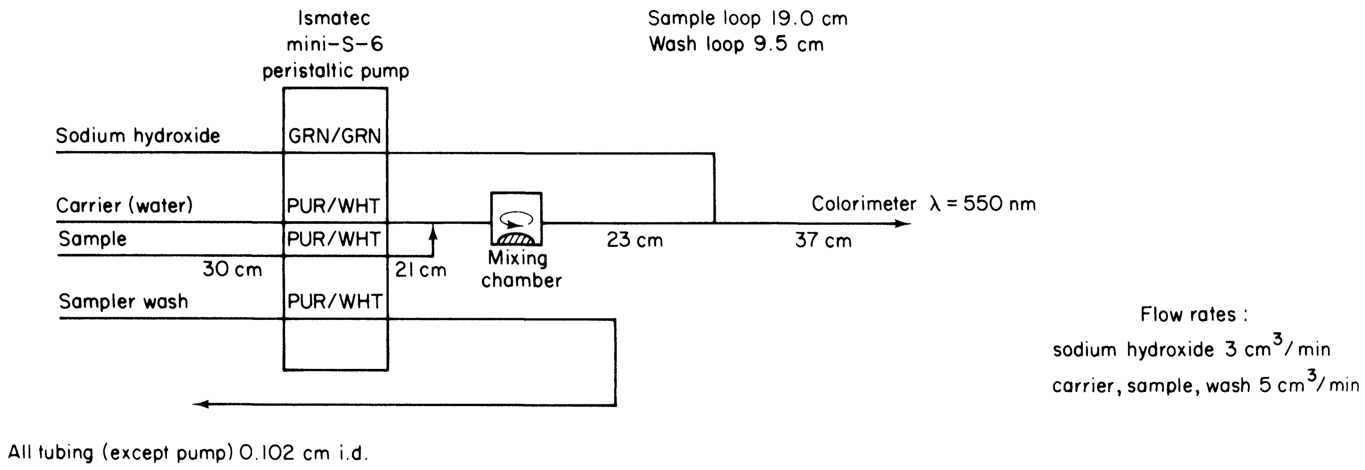

*Figure 2. Pumping arrangement of the system.*

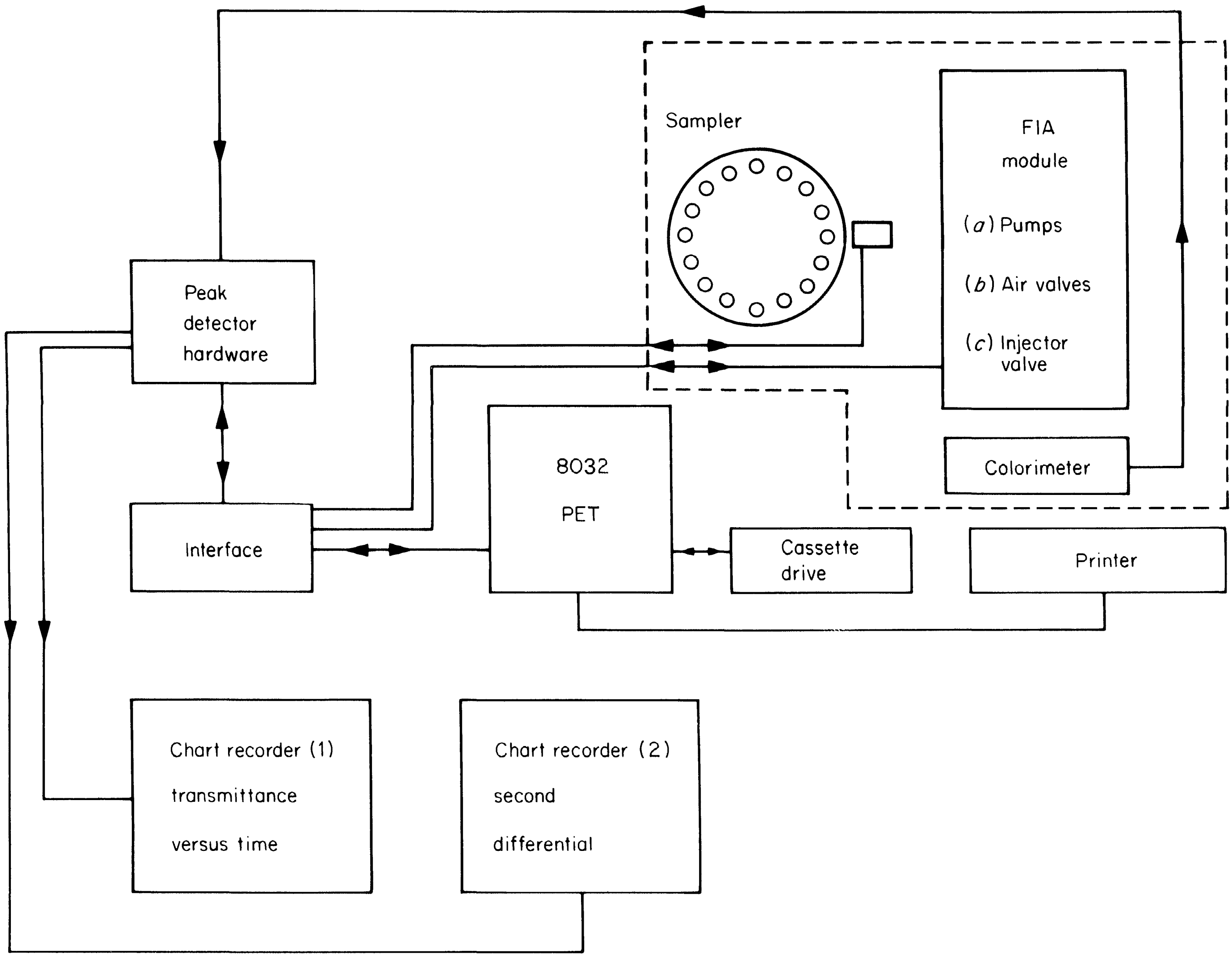

*Figure 3. Microcomputer control.*

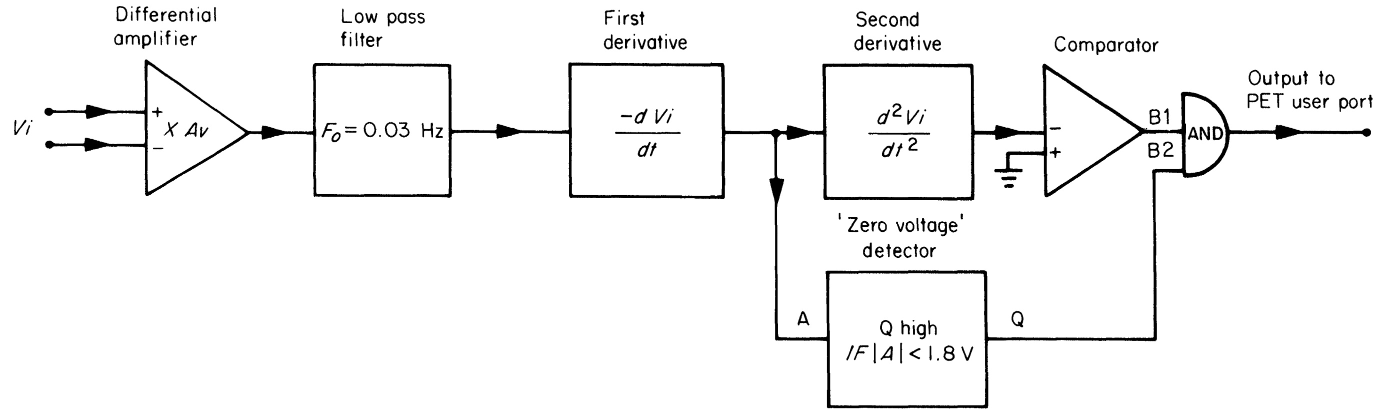

*Figure 4. Schematic diagram of in-house constructed hardware for timing the distance between the points of inflection of the transmittance/time curves.*

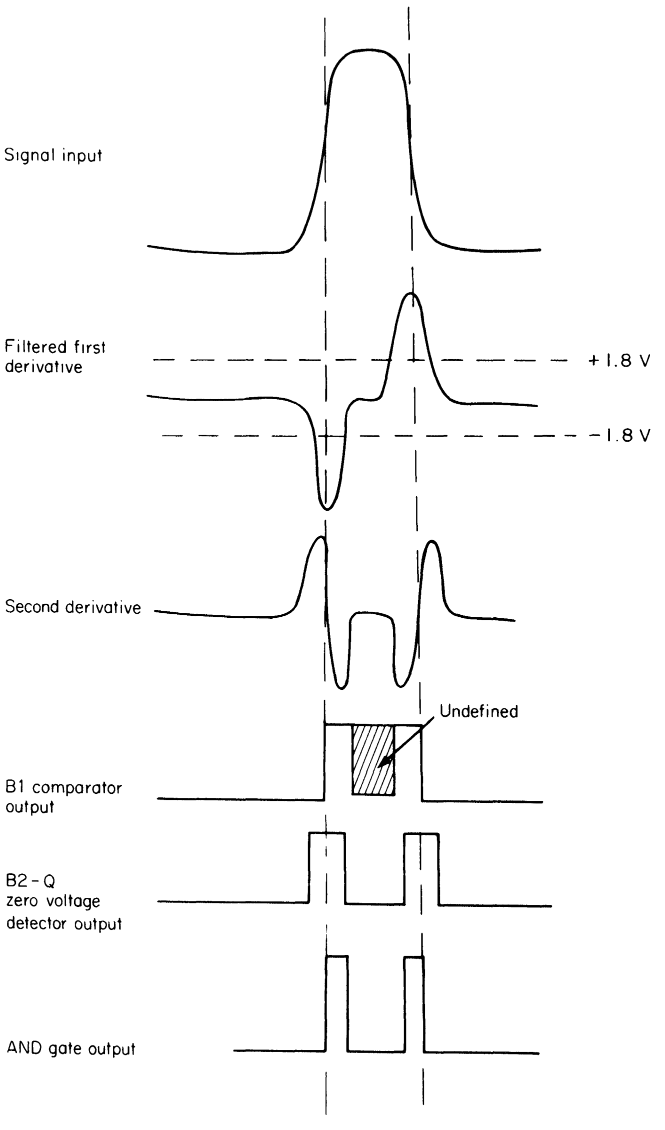

*Figure 5. Signals at the output of various stages shown in figure 4.*

Values for the slope, intercept, residual standard deviation and correlation coefficient are calculated and printed-out (figure 4). Subsequent values for *ti* (*N*) corresponding to the samples are fitted to the calibration line and a value for the concentration is calculated and printed out until the last sample is reached.

### Results

Table 1 shows a comparison between the results obtained with the manual method and those obtained with the automatic method. The results of the manual titration are normally quoted in millilitres of 0·1M sodium hydroxide. For the purpose of

*Table 1*

| *TA* manual | *TA* automatic | *d* | *TA* manual | *TA* automatic | *d* |
|---|---|---|---|---|---|
| 1·5 | 1·50 | 0 | 2·6 | 2·97 | 0·4 |
| 3·0 | 2·77 | −0·2 | 2·7 | 2·17 | −0·5 |
| 6·9 | 6·25 | −0·6 | 2·1 | 3·86 | 1·8 |
| 3·6 | 3·83 | 0·2 | 27·3 | 22·55 | −4·7 |
| 5·5 | 5·05 | −0·4 | 3·4 | 3·05 | −0·3 |
| 3·6 | 3·05 | −0·5 | 3·5 | 3·25 | −0·2 |
| 2·2 | 2·30 | 0·1 | 2·3 | 2·70 | 0·4 |
| 7·5 | 7·90 | 0·4 | 5·1 | 5·00 | −0·1 |
| 4·3 | 4·70 | 0·4 | 2·8 | 3·00 | 0·2 |
| 3·0 | 2·80 | −0·2 | 2·9 | 2·50 | −0·4 |
| 4·8 | 4·80 | 0 | 3·0 | 2·70 | −0·3 |
| 2·7 | 2·30 | −0·4 | 4·2 | 3·60 | −0·6 |
| 8·6 | 7·50 | −1·1 | 2·8 | 2·20 | −0·6 |
| 2·9 | 3·40 | 0·5 | 1·3 | 1·80 | 0·5 |
| 2·7 | 2·85 | 0·1 | | | |

*TA* = Total acidity.

$$t=\frac{\bar{d}}{s/\sqrt{N}}=\frac{-0{\cdot}22155}{0{\cdot}1900786}=-1{\cdot}1656$$

$\bar{d}$ is the mean of the differences beteeen *TA* manual and *TA* automatic. *s* is the standard deviation and *N* the number of degrees of freedom.

$$t_{0\cdot025,28}=2{\cdot}05$$

$t_{0\cdot025,28}$ refers to the tabulated value at 95% confidence level for 28 degrees of freedom.

```

1 d1$="000040":d2$="000250":d3$="000245"

5 n=1:poke 59459,15

8 input "no. of samples";t

10 dim t(10+t):dim s(10+t):dim c(10+t)

15 poke 59471,3 :ti$="000000"

16 ti$="000000"

20 if (peek(59471) and 16 )=0   then 35

25 if ti$=d1$ then poke 59471,0

27 if ti$=d2$ then poke 59471,3

30 if ti$>d3$ then 55

32 goto 20

35 g=ti

36 if (peek(59471) and 16)=0 then 36

40 if (peek(59471) and 16 )=0   then 48

42 if ti$=d1$ then poke 59471,0

43 if ti$=d2$ then poke 59471,3

44 if ti$>d3$ then 55

47 goto 40

48 if (peek(59471) and 16)=16 then 51

49 if ti$>d3$ then 55

50 goto 48

51 t(n)=(ti-g)/60

53 goto 40

55 s(n)=(int(100*t(n)+.5))/100

60 print ""

63 n=n+1

65 if s(n-1)<s(10) then gosub 810

70 print"peak no.";n-1;"detected ";s(n-1)

72 if ti$<d1$ then 72

75 if n=11+t then 85

77 if ti$<d2$ then 77

80 goto 15

85 print

90 open 1,4

95 print#1,

100 print "          standards": print

105 print#1,"         standards"

110 print "conc.","actual","predicted"

120 print#1,"actual","predicted"

140 print

200 for n=1 to 10

210 p=p+t(n)

220 read a

230 b=b+a

240 next n

250 q=p/10

260 c=b/10

270 restore

280 for n=1 to 10

290 y=y+(t(n)-q)^2

300 read a

310 x=x+(t(n)-q)*(a-c)

315 z=z+(a-c)^2

320 next n

330 m=x/z

340 i=q-m*c

350 restore

360 for n=1 to 10

370 read a

380 l(n)=int(100*(m*a+i)+.5)/100

390 print#1,s(n),l(n)

400 print a,s(n),l(n)

410 d=d+(t(n)-m*a-i)^2

420 next n

430 r=sqr(d/8)

435 f=x/sqr(z*y)

440 print

450 print#1,

460 print "slope =";m

470 print#1,"slope =";m

480 print

490 print#1,

500 print "intercept =";i

510 print#1,"intercept =";i

520 print

530 print#1,

540 print "residual standard deviation =";r

550 print#1,"residual standard"

560 print#1,"deviation =";r

580 print#1,

590 print "correlation coefficient =";f

600 print#1,"correlation coefficient"

610 print#1,"   =";f

620 if t=0 then 800

650 print

660 print#1,

665 print "   samples"

670 print#1,"       samples"

680 print

700 print "no.","conc."

710 print#1,"no.","conc."

720 print

730 for n=11 to 10+t

740 c(n)=(t(n)-i)/m

750 print n-10,int(c(n)*100000+.5)/100000

760 print#1,n-10,

770 print#1,int(c(n)*100000+.5)/100000

780 next n

800 stop

810 print "Warning - sample concentration

    outside range of standards"

820 return

900 data 1,1.125,1.301,1.602,2

910 data 2.125,2.301,2.602,3,3.125

```

*Figure 6. Commodore PET 8032 software.*

comparison, the results obtained with the automatic method have been translated into a corresponding volume of 0·1M NaOH. A paired comparison test was performed on the data of table 1 to test the mean values obtained from the method. The test considers the hypothesis of no difference between the two methods and the hypothesis that there is a difference ($t_{calc} = -1{\cdot}17$, $t_{0{\cdot}025,28} = 2{\cdot}05$). The result of the test is that there is no significant difference between the methods at 95% confidence levels. The average difference between the manual results and the FIA results was −0·22 ml.

Results from replicate analyses of 15 samples were used for a one-way analysis of variance (table 2). This showed that the method has a repeatability standard deviation of 0·69.

### The effect of acidity, colour and density on peak width

The variation of peak width with acidity for a set of 10 colourless standards is shown in figure 7. Each series of analyses is performed by preceding the beer samples with 10 standards. The statistical evaluation of linearity for the standards is summarized by the slope and intercept. The residual standard deviation, *s*,

*Table 2*

| Source of variation | Sum of squares (1) | DF (2) | Mean square (1)/(2) |
|---|---|---|---|
| Between samples | 848·3847 | 14 | 60·5989 |
| Between replicates | 7·0450 | 15 | 0·46967 |
| Total | 855·4297 | 29 | |

Repeatability standard deviation = 0·6853.
DF = Degrees of freedom.

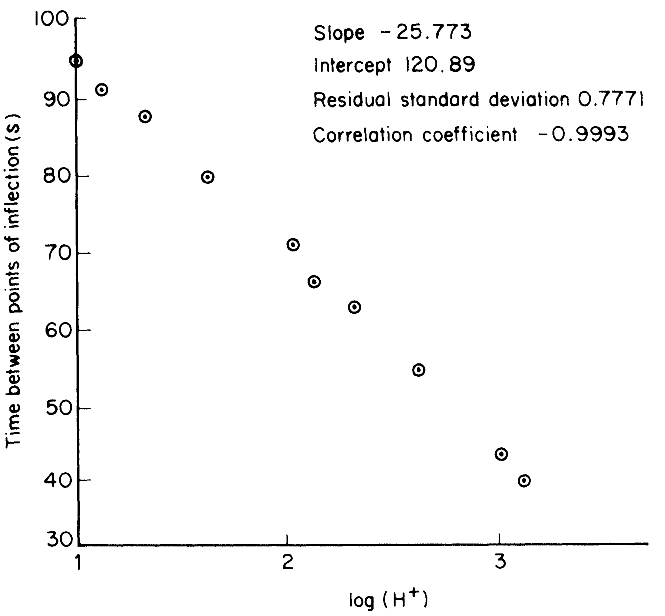

*Figure 7. Plot of time between points of inflection versus logarithm of hydrogen ion concentration.*

and the correlation coefficient, $r$, are also found. These values, and values for the slope and intercept of the best fit, follow immediately behind the values of $ti\,(N)$ for each standard on the hard-copy print-out.

The best line is calculated through the centroid of the data and is the best fit that can be obtained through the points. The fit minimizes the distances between the actual and predicted values. The residual standard deviation represents the error involved in the fit and is found from the equation:

$$s=\sqrt{\sum ti(N)-ti(N)^1)^2/N-2}$$

where $ti(N)$ is the actual value of the time between inflection points and $ti(N)^1$ is the predicted value; $(N-2)$ represents the number of degrees of freedom. The correlation coefficient is a measure of the degree of association between $ti(N)$ and $\log\,[H^+]$ for the 10 standards. It is given by the equation:

$$r=Sxy/\sqrt{Sxx\cdot Syy}$$

$$Sxx=\sum(ti(N)-\overline{ti(N)})^2$$

$$Syy=\sum(\log[H^+]-\overline{\log[H^+]})^2$$

$$Sxy=\sum(ti(N)-\overline{ti(N)})(\log[H^+]-\overline{\log[H^+]})$$

Betteridge [7] has observed that the degree of mixing of a sample zone with a carrier stream is a function of the viscosity of the sample solution. By injecting glycerol/water mixtures into a carrier stream of water containing dye, he was able to show that greater spread of dye into the sample zone occurred at lower sample viscosity.

Figure 8 shows the variation in $ti(N)$ with differing concentrations of sucrose over the range 0% to 10% at a constant acidity of 0·001 M $H^+$. This range of sucrose concentrations represents a variation in relative viscosity $\eta/\eta_0$ of 1·333 (where $\eta$ is the absolute viscosity of the sucrose solution at 20°C and $\eta_0$ is the absolute viscosity of water at 20°C) [8]. Experiments measuring the time taken to flow through an orifice from a constant hydrodynamic head for several beer samples showed that this range of sucrose concentrations was representative. Over this concentration range a change in $ti(N)$ of about 2 s occurred. (Betteridge considered sample zones varying in relative viscosity between 1·5 and 1760.)

Figures 9 and 10 show the effect on the peak shape of changing from a colourless standard to a coloured one. The major effect is the loss of the flat tops and the generation of a dip towards the base line. Figure 11 shows a definite positive correlation between the degree of peak distortion and caramel content.

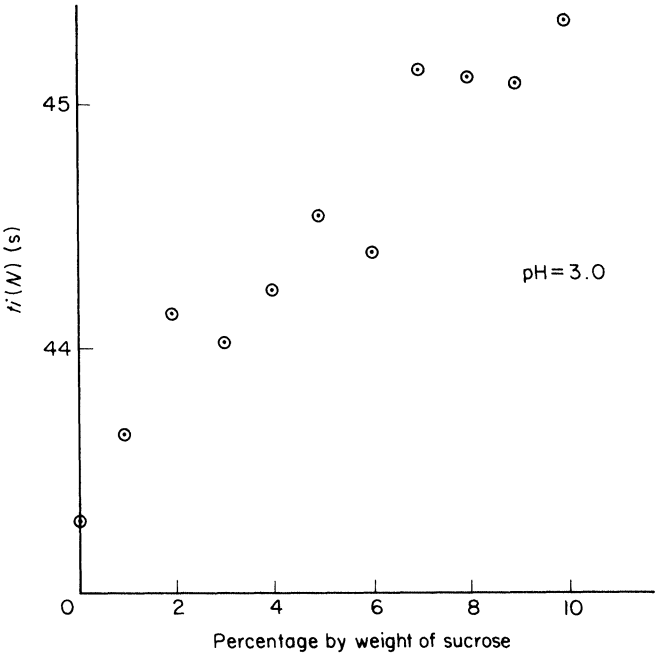

*Figure 8. Variation in time between points of inflection with differing concentrations of sucrose.*

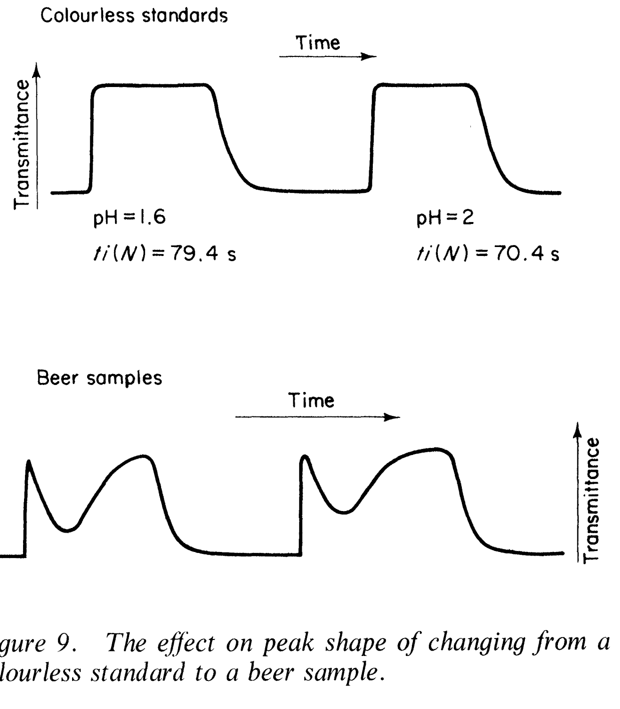

*Figure 9. The effect on peak shape of changing from a colourless standard to a beer sample.*

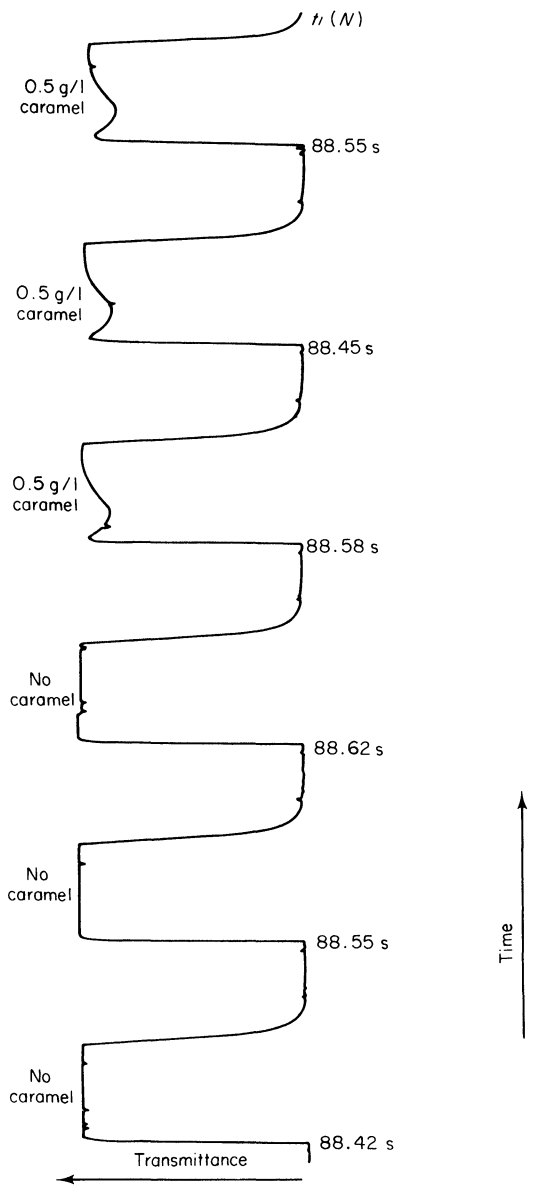

| ti (N) s | Concentration of caramel (grade W) | pH |
|---|---|---|
| 88.42 | 0 | 1 |
| 88.55 | 0 | 1 |
| 88.62 | 0 | 1 |
| ti (N) 88.53 | | |
| 88.58 | 0.5 g/L | 1 |
| 88.45 | 0.5 g/L | 1 |
| 88.55 | 0.5 g/L | 1 |
| ti (N) 88.53 | | |

*Figure 10. The effect on the peak shape of changing from a colourless standard to a coloured one.*

### Discussion and conclusions

The apparatus described in this paper has been successfully operated by staff not previously familiar with analytical automation or microcomputers, but who were practised at performing the manual acid/base titration. The choice of a colorimetric

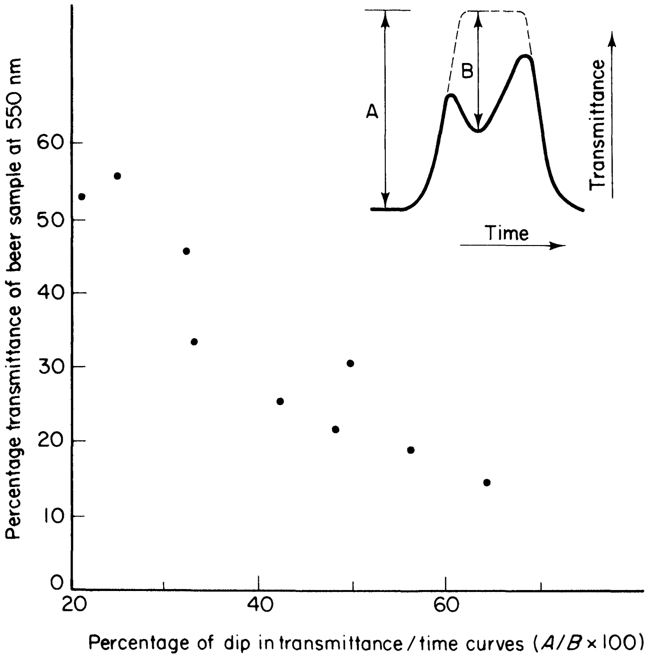

*Figure 11. Plot of degree of peak distortion versus caramel content.*

detection system was made not only on the grounds that a pH sensing electrode would be harmed by long-term usage in beer samples, but also that the results obtained with the automatic method had to be directly relatable to the manual method.

Aside from the forementioned problems associated with density and colour variations of samples, the apparatus is a prototype and has some shortcomings. The combination of hardware depicted in figure 3 is cumbersome and has the disadvantage of requiring an air supply to operate the injection system. The cumbersome nature of the apparatus is not improved by the multi-module interfacing associated with the microcomputer.

The cost saving achieved by employing a peristaltic pump primarily designed for two channels and used to drive four channels is satisfactory. Care needs to be observed, however, when commencing pumping with dry pump tubes. Other difficulties exist in the preparation and storage of acid standards covering a suitable range. Although figure 3 has included two chart recorders, these have been dispensed with in the routine analytical method.

### Acknowledgements

The authors are grateful to the Government Chemist for permission to publish this paper.

### References

1. Ramsing, A. V., Ruzicka, J. and Hansen, E. H., *Analytica Chimica Acta*, **129** (1981), 1.
2. Stewart, K. K., *Talanta*, **28** (1981), 789.
3. Ramsing, A. V. *et al.*, *Analytica Chimica Acta*, **129** (1981), 1.
4. Pardue, H. L. and Fields, B., *Analytica Chimica Acta*, **124** (1981), 39.
5. Stewart, K. K. and Rosenfield, A. G., *Journal of Automatic Chemistry*, **3** (1981), 30.
6. Sawyer, R., Dixon, E. J., Lidzey, R. G. and Stockwell, P. B., *Analyst*, **95** (1970), 957.
7. Betteridge, D., *Talanta*, **23** (1976), 409.
8. *Handbook of Chemistry and Physics* (CRC Press, 1979), 60, 270.

